# Membrane Microreactors for the On‐Demand Generation, Separation, and Reaction of Gases

**DOI:** 10.1002/chem.202001942

**Published:** 2020-09-11

**Authors:** Christopher A. Hone, C. Oliver Kappe

**Affiliations:** ^1^ Center for Continuous Flow Synthesis and Processing (CCFLOW) Research Center Pharmaceutical Engineering GmbH (RCPE) Inffeldgasse 13 8010 Graz Austria; ^2^ Institute of Chemistry University of Graz, NAWI Graz Heinrichstrasse 28 8010 Graz Austria

**Keywords:** flow chemistry, gas–liquid, membrane, microreactors, tube-in-tube reactor

## Abstract

The use of gases as reagents in organic synthesis can be very challenging, particularly at a laboratory scale. This Concept takes into account recent studies to make the case that gases can indeed be efficiently and safely formed from relatively inexpensive commercially available reagents for use in a wide range of organic transformations. In particular, we argue that the exploitation of continuous flow membrane reactors enables the effective separation of the chemistry necessary for gas formation from the chemistry for gas consumption, with these two stages often containing incompatible chemistry. The approach outlined eliminates the need to store and transport excessive amounts of potentially toxic, reactive or explosive gases. The on‐demand generation, separation and reaction of a number of gases, including carbon monoxide, diazomethane, trifluoromethyl diazomethane, hydrogen cyanide, ammonia and formaldehyde, is discussed.

## Graphical abstract

**Go with the flow**: Gas–liquid transformations are very important for organic synthesis. However, gases can be very challenging to use at laboratory scale. Continuous flow membrane reactors enable the safe on‐demand laboratory‐scale generation, separation, and reaction of gases without the need for distillation or gas cylinders.

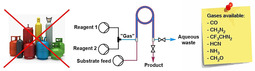

## Introduction

Gas–liquid transformations are very important for organic synthesis. Whether a chemical is widely used will depend on the easiness to source, transport, store and handle the chemical. It is becoming increasingly difficult to transport dangerous and toxic gases due to ever growing restrictions.^1^ Furthermore, some gases are simply too reactive or short‐lived to be produced, stored and transported for later use.^2^ Users may be hesitant to use a gas due to safety concerns, or perhaps lack the necessary experience or infrastructure to support their use. In addition, more specialized and expensive equipment is required for accessing higher pressures (e.g., autoclave apparatus). Whilst the use of gases on an industrial scale provides little hindrance, at a laboratory scale these challenges are unfortunately responsible for the underuse of toxic and flammable gases within many organic chemistry laboratories.^3^ The majority of alternatives to the use of gases are often more expensive, atom inefficient reagents (“gas surrogates”), that are not a viable option for use in large scale manufacturing. Thus process chemists are forced to swap these protocols with conventional gaseous reagents during scale‐up studies, which adversely impacts the time‐to‐market due to the cost and time associated with the re‐development.

## Conventional Approaches for Handling Gases

There are a number of approaches used for the introduction of a gas into an organic reaction. The exact approach used depends on a number of aspects, including the properties of the gas, the reaction conditions needed for the organic transformation, the scale and the capabilities of the available equipment.

A common approach at a laboratory scale is to simply affix a pre‐filled balloon to a round‐bottom flask to provide a gas at atmospheric pressure. This approach is commonly used, but is unsafe if the balloon suddenly bursts and investigation of pressure effects is not possible. A syringe can also be pre‐loaded with a gas and used to introduce the gas at a controlled flow rate. A more elegant method for the introduction of a gas directly from a cylinder is by use of a mass flow controller (MFC) to regulate flow rate. However, the installation of gas cylinders within a laboratory can be time consuming and cause accommodation issues, such as the requirement for a fire resistant storage cabinet. For gases with a low vapor pressure, a common method is to condense the gas into the liquid phase for use in a reaction.^4^ The condensation of a gas can be highly hazardous due to flammability issues. Gases can also be formed in situ or ex situ from solid or liquid reagents, known as gas surrogates.^5^, ^6^ Some gases are highly reactive and therefore need to be prepared on‐site and only shortly before use. Dangerous purification operations (e.g., distillation) are often necessary for isolation of these gases with sufficient purity. The main limitation of an in situ approach is that there are often chemical compatibility issues between the gas forming reaction and the organic transformation consuming the gas. In many instances, the conditions necessary for the generation of a gas uses acidic or basic aqueous conditions, therefore making it difficult to produce a gas in an anhydrous manner, which is critical for the performance of many organic reactions.

To overcome the compatibility issues, Skrydstrup and co‐workers devised a two‐chamber batch glassware system for the ex situ generation of gas.^7^ The system comprises of two separate chambers which are connected to allow the passage of gas from the chamber for gas generation to the chamber for gas consumption. This approach has the limitation that a gas can accumulate within the system if not consumed at a sufficient rate and that it can only be operated at low pressures.

## Gas–Liquid Membrane Microreactors

**Figure 1:**
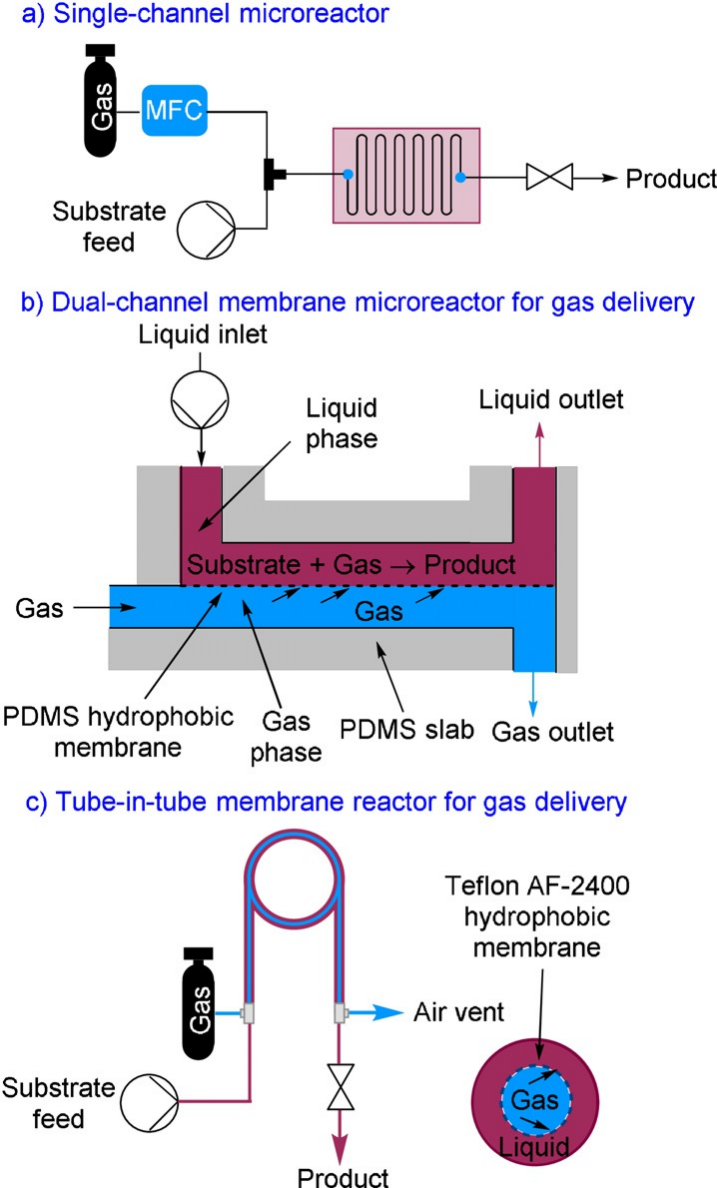
Gas delivery within micro‐ and tubular reactors: a) single‐channel microreactor, b) dual‐channel microreactor, and c) tube‐in‐tube reactor.

Over recent years, microreactors and continuous flow technologies have emerged as an enabling tool for the safe handling of hazardous chemistry.^8^, ^9^ In particular, for accessing “forgotten” and “forbidden” chemistry which cannot be accessed under conventional batch conditions.^8^, ^9^, ^10^ The small internal dimensions of continuous flow reactors provide a high surface‐to‐volume ratio, resulting in enhanced heat and mass transfer characteristics.^11^ The small internal volume ensures only a small chemical inventory is handled at any one time. The on‐site production and consumption of a hazardous chemical within a single integrated unit improves the inherent safety and eliminates transportation and storage of highly reactive and hazardous reagents.^2^ Furthermore, the inclusion of an in‐line quench within a flow setup or immediately afterward avoids the accumulation of a highly reactive chemical. Due to the merits of flow reactors, they have been widely used for performing gas–liquid reactions.^12^ Single‐channel microreactors are most commonly used for gas–liquid reactions (Figure 1 a). Gas–liquid reactions within these systems employ a biphasic flow regime, most commonly segmented (Taylor) flow, which facilitates rapid mixing and mass transfer.

Recently, membrane microreactors have gained significant attention as efficient gas–liquid contactors for performing organic transformations.^13^ The membrane separates two adjacent channels, typically with one containing a liquid phase and the other a gas phase. A semi‐permeable membrane with a very large surface area allows the selective passage of gases and low molecular weight compounds from one side to the other. The gas is rapidly consumed by the substrate in the second channel. The membranes used display hardly any liquid permeability and are selected to display broad chemical resistance. The nature of the contacting method ensures the process is inherently safe because the liquid phase and gas phase are in different channels, and the gas is fully dissolved in the liquid phase, thus flammable organic solvents are never in the presence of a gas (vapor) phase.^9^, ^14^

Kim and co‐workers developed a dual‐channel microreactor strategy for performing gas–liquid transformations (Figure 1 b).^15^ The microreactor is fitted with a hydrophobic poly(dimethylsiloxane) (PDMS) membrane (45 μm thickness) which allows diffusion of gases but is impermeable to the other reaction components. A gas flows along one channel passing through the membrane into the second channel containing the liquid phase for the organic transformation. The system was first reported for reactions involving O_2_.^16^ Subsequently, the reactor was modified to a three‐channel microreactor so that the gas could be introduced from both sides of the liquid channel.^17^ In this system, the fabrication is simpler and the effective interfacial area is doubled.

About the same time, Ley and co‐workers pioneered the tube‐in‐tube membrane reactor for the loading of gases for use in organic reactions (Figure 1 c).^18^ The inner tubing is manufactured from a gas‐permeable and hydrophobic fluoropolymer, Teflon AF‐2400. Teflon AF‐2400 is a copolymer of tetrafluoroethylene and perfluorodimethyldioxolane.^19^ The outer tubing is either manufactured from plastic or stainless steel tubing depending on the system requirements. Typically, the system is operated with the gas in the inner tube and the liquid phase in the outer tubing. The reactor has been successfully demonstrated for a number of gases including CO, H_2_, CO, CO_2_, O_2_, O_3_, NH_3_, fluoroform, and ethylene.^20^ There are two modes of operation for the tube‐in‐tube reactor, either: (1) a liquid‐phase is pre‐saturated with a gas within the tube‐in‐tube system prior to a subsequent reaction within a second reactor; or (2) the loading of gas to the liquid channel and organic transformation are performed simultaneously within the tube‐in‐tube system. A number of versions of the tube‐in‐tube reactor are commercially available.^21^

## On‐Demand Generation, Separation & Reaction of Gases

**Figure 2:**
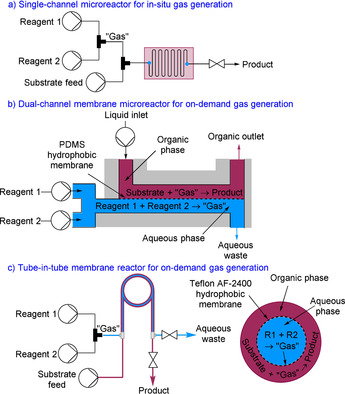
On‐demand gas generation, separation and reaction within micro‐ and tubular reactors: a) single‐channel flow reactor, b) dual‐channel microreactor, and c) tube‐in‐tube reactor.

In this article, we present the concept of the on‐demand generation, separation and reaction of gases within continuous flow membrane systems (Figure 2). A membrane is necessary to ensure the gas generation and consumption occur in two separate channels. There are examples reported that generate a gas from reagents within a single‐channel microreactor, but this approach relies on compatibility between the conditions for gas generation and gas consumption, and also complicates post‐reaction processing.^22^

The membrane microreactors described above can also be used for this purpose. A gas can be generated from inexpensive and readily available reagents within one channel. These reagents should be safer to handle than the corresponding gas formed. The generation of a gas can be carefully controlled through the cautious manipulation of the reagent flow rates to produce the required stoichiometry for reaction. The release of the gas within the system can be controlled to avoid a potential buildup of pressure.

Subsequently, the gas selectivity diffuses through the membrane into an adjacent chamber. The membrane is generally impermeable to all other components. The second channel contains the chemistry for the main organic transformation. The use of incompatible conditions for the gas generation and consumption is possible, for example acidic conditions in one channel and basic in the second channel, or aqueous conditions in one channel and anhydrous conditions in the other channel. Thus, anhydrous gas can be prepared without dangerous purification operations (e.g., distillation). The gas is consumed as it is formed which minimizes the risk of accumulation, therefore the approach is very safe because only a small quantity of gas is present within the system at any one time. The generation of a gas, its separation and organic transformation are fully contained within a closed system preventing any exposure to the operator. It also completely obviates the need for gas cylinders.

Herein, a number of examples demonstrating the use of membrane flow technologies for the successful continuous generation, separation and reaction of gases are discussed. The relative strengths and weaknesses of the strategies are covered.

### Carbon monoxide (CO)

Carbon monoxide (CO) is a highly valuable C1 building block due to the number of carbonylation reactions available to the synthetic chemist.^23^ CO is a colorless, odorless and tasteless gas. It is highly poisonous and flammable, thus it is underused for organic synthesis.

**Scheme 1:**
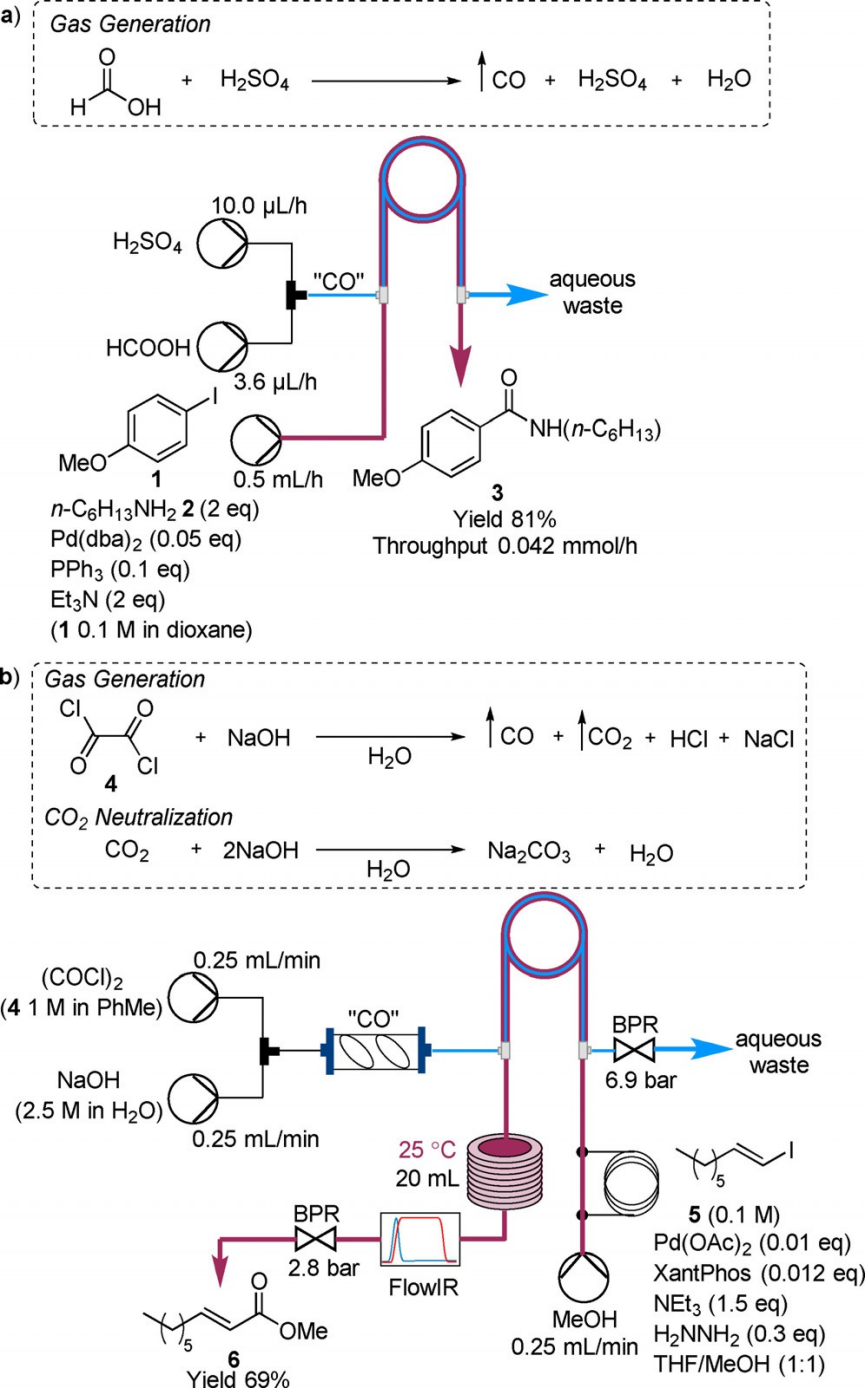
On‐demand generation, separation and reaction of CO within a tube‐in‐tube reactor for: a) Pd‐catalyzed aminocarbonylatio and b) Pd‐catalyzed alkoxycarbonylation. Pd(dba)_2_=bis(dibenzylideneacetone)palladium, Xantphos=4,5‐bis(diphenylphosphino)‐9,9‐dimethylxanthene.

Perhaps the earliest example using the tube‐in‐tube reactor for on‐demand gas generation, separation and reaction was reported by Ryu and co‐workers (Scheme 1 a).^24^ In this report, CO was generated within the inner tube through the dehydration of formic acid (HCOOH) with sulfuric acid (H_2_SO_4_). The liberated CO then passed through the membrane to the outer tube for the main organic transformation. The main organic reaction was a palladium‐catalyzed Heck aminocarbonylation of 4‐iodoanisole (**1**) with *n*‐hexylamine (**2**) to form the amide **3**. The protocol demonstrated that CO could be continuously generated and consumed with amide **3** afforded in 81 % yield within 3 h residence time. This particular example highlights the benefit of using membrane technology as the acidic conditions to liberate CO could be used concomitantly with the basic conditions used in the carbonylation reaction. The study was an excellent proof‐of‐concept, but the conditions employed are very limited by their throughput (0.042 mmol h^−1^).

More recently, Ley and co‐workers reported an alternative reaction system for generating CO within the tube‐in‐tube reactor (Scheme 1 b).^25^ Oxalyl chloride (COCl)_2_ (**4**) was hydrolyzed by sodium hydroxide (NaOH) within the outer channel of the tube‐in‐tube reactor. The use of an Omnifit column (0.68 mL) with two magnetic stirrer bars prior to the tube‐in‐tube reactor was necessary to adequately mix the toluene phase containing COCl_2_ and the aqueous NaOH phase. CO then passed through the membrane into the inner tube for the main organic transformation which was flowing counterflow to the CO generating stream. A FlowIR spectrometer was used in‐line for reaction monitoring. CO_2_ was observed by FTIR to pass through the membrane under certain conditions, therefore the ratio of NaOH to COCl_2_ was increased to neutralize the CO_2_ formed. The system was optimized on the Pd‐catalyzed methoxycarbonylation of vinyl iodide **5** (Scheme 1 b). The alkoxycarbonylation was demonstrated on a number of vinyl and aryl iodides (eight examples), and aminocarbonylation (two examples). The potential scalability of the system was demonstrated with a scale‐out long run for 320 min operation time, with a throughput of 1.43 mmol h^−1^ achieved.

### Diazomethane (CH_2_N_2_)

Diazomethane (CH_2_N_2_) is a highly valuable reagent for the introduction of a methyl or a methylene group into a molecule.^26^ Diazomethane is used for the preparation of methyl esters from their corresponding carboxylic acids, homologation of ketones or carboxylic acids (Arndt–Eistert reaction) and cyclopropanation reactions. However, diazomethane is a potent carcinogen, extremely toxic, odorless yellow gas. In general, diazomethane is generated and purified by co‐distillation with diethyl ether which is associated with a certain safe risk. It has a high explosive potential and the vast majority of diazomethane explosions occur during the distillation process. Consequently, diazomethane is hardly ever used for the industrial production of chemicals due to safety concerns.^26^, ^27^ Anhydrous diazomethane can be prepared without the need to distill through the application of membrane microreactors.

**Scheme 2:**
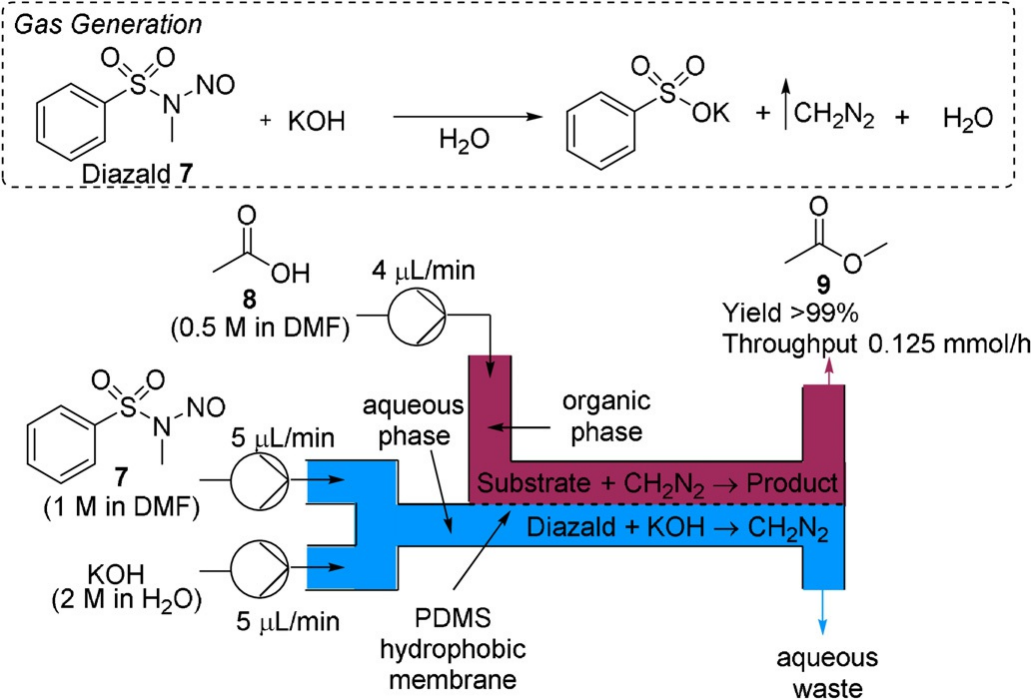
Dual‐channel microreactor for the in situ generation, separation and reaction of diazomethane for the methylation of acetic acid.

Kim and co‐workers reported the use of the dual‐channel microreactor for the in situ generation, separation and reaction of diazomethane (Scheme 2).^28^ Diazomethane can be generated from the base‐mediated decomposition of *N*‐methyl‐*N*‐nitroso‐*p*‐toluenesulfonamide (Diazald, **7**). In this flow configuration, **7** quickly reacts with KOH to generate diazomethane in the bottom channel, and then diffuses through the PDMS membrane to the upper channel for immediate consumption by reaction with the substrate. The system was successfully optimized for the methylation of acetic acid (**8**) to afford methyl acetate (**9**) with a moderate throughput of 0.125 mmol h^−1^. The microreactor has a very small internal volume (60 μL) which improves the inherent safety, but limits throughput. The methylation of phenol (Table 1, entry 1), methylation of benzaldehyde (entry 2) and the Arndt–Eistert reaction of benzoic chloride (entry 3) were successfully demonstrated using the system.

**Table 1:** Scope for reactions performed in the dual‐channel microreactor.^[a]^

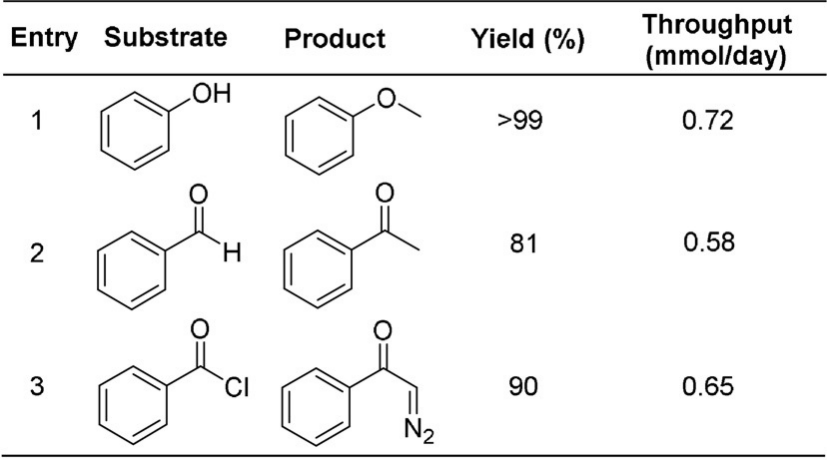

[a] Diazomethane was generated in the bottom channel by flowing solutions of Diazald (1.0 m in DMF) and KOH (2.0 m in water containing 0.01 % aliquat 336) at the same flow rate. Substrates were introduced to the top channel in DMF (0.5 m solution). Organic flow rate=1 μL min^−1^, KOH+diazald flow rate=4 μL min^−1^.

**Scheme 3:**
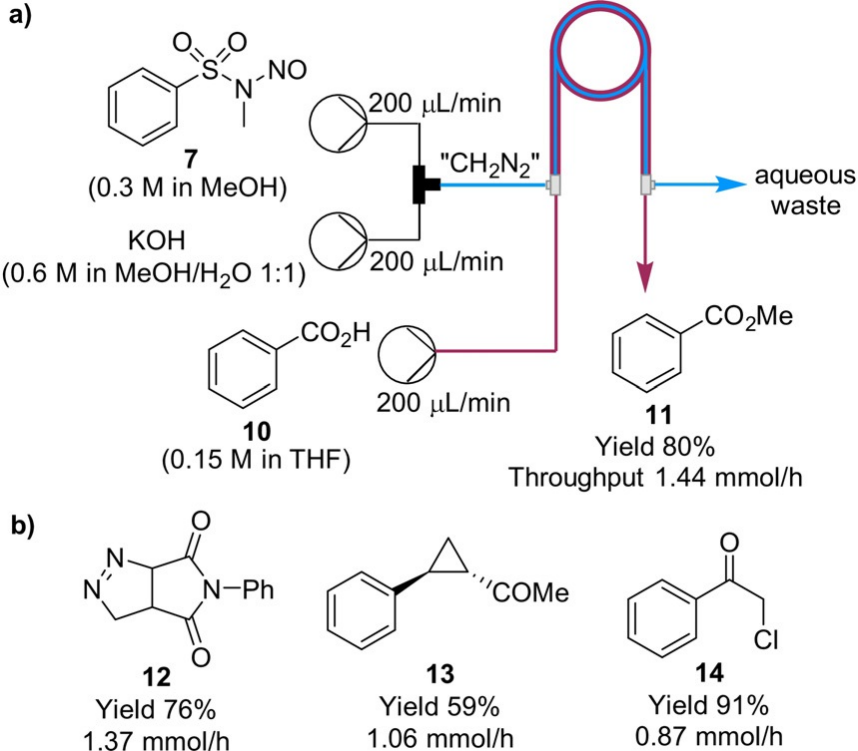
a) On‐demand generation, separation and reaction of diazomethane within a tube‐in‐tube reactor for the methylation of benzoic acid. b) Products from a [3+2] cycloaddition, Pd‐catalyzed cyclopropanation of an alkene, and an Arndt–Eistert reaction.

Along similar lines to the CO formation, CH_2_N_2_ can also be generated from KOH and Diazald (**7**) within the inner tubing of a tube‐in‐tube reactor, with CH_2_N_2_ diffusing through the membrane to be consumed within the substrate‐carrying outer channel (Scheme 3).^29^ Our group optimized the system for the methylation of benzoic acid (**10**) (Scheme 3 a). The conditions were applied for the methylation of a number of nucleophiles (six examples). Subsequently, the configuration was also demonstrated for a [3+2] cycloaddition, Pd‐catalyzed cyclopropanation and an Arndt–Eistert reaction (Scheme 3 b). We also used the system in the context of making precursors for antiretroviral drugs in a fully telescoped flow manner.^30^ Various *N*‐protected amino acids were converted into their corresponding α‐halo ketones with good yields for an important step in the synthesis (eight examples). Koolman and co‐workers also reported the synthesis of cyclopropyl boronic esters by Pd‐catalyzed cyclopropanation.^31^ In this case, CH_2_N_2_ was generated and separated within the tube‐in‐tube reactor to pre‐saturate the organic phase prior to the introduction of the other reagents for the cyclopropanation reaction, which occurred in a subsequent reactor coil reaction (14 examples).

**Scheme 4:**
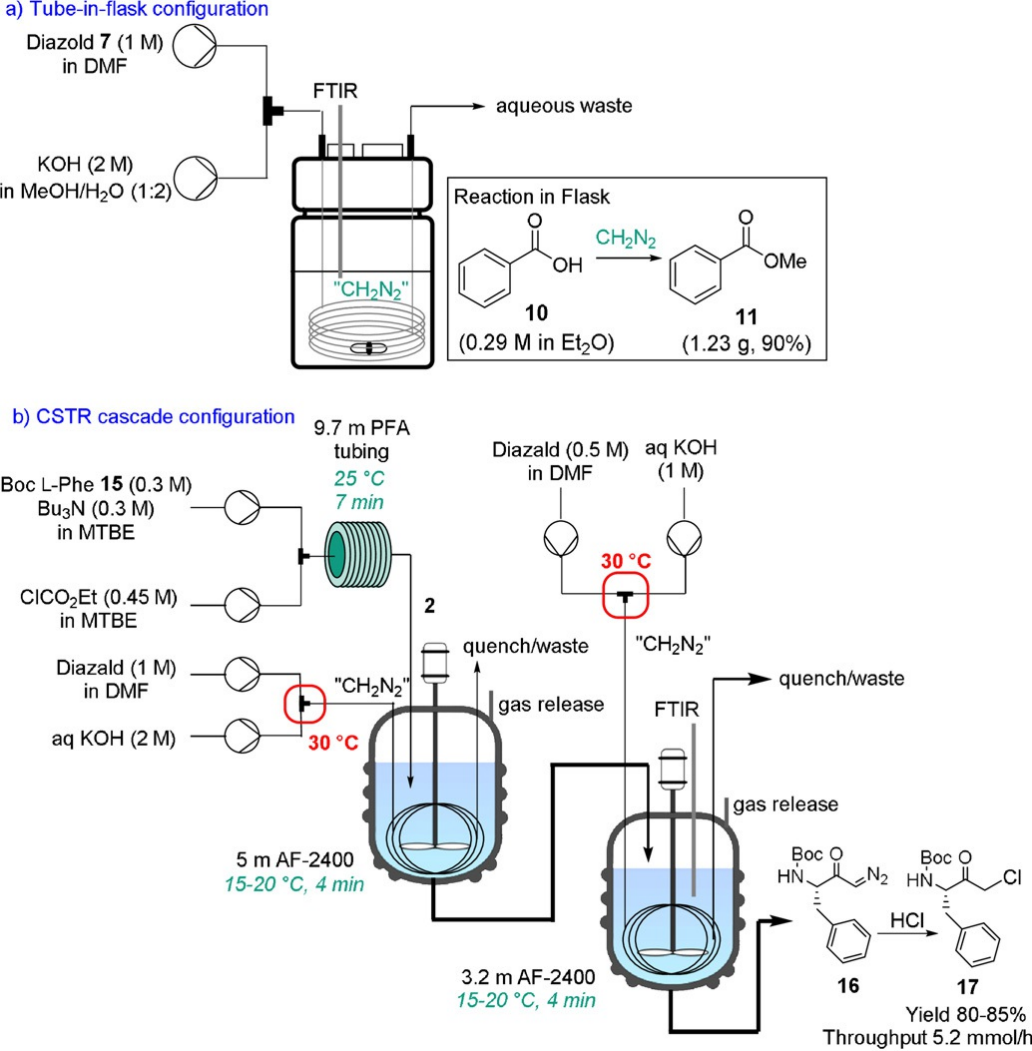
On‐demand diazomethane generation with different reactor setups: a) Tube‐in‐flask configuration and b) CSTR cascade for diazoketone **16** synthesis.

A drawback of the tube‐in‐tube reactor is that it is limited to a CH_2_N_2_ throughput of approximately 1.25 mmol h^−1^. A subsequent development was the tube‐in‐flask reactor.^32^ In this configuration, the membrane is coiled inside a glass flask, CH_2_N_2_ is generated continuously within the membrane, and CH_2_N_2_ diffuses through the membrane into a flask filled with substrate and solvent (Scheme 4 a). The tube‐in‐flask reactor also allows the organic transformation to contain solids. Although not yet commercially available, the tube‐in‐flask reactor can be assembled from commercially available parts within ∼1 h.^33^ Furthermore, simple parallelization of the membranes enables a moderate throughput of material to be achieved (CH_2_N_2_ at ≈42.8 mmol h^−1^). However, throughput is still limited due to safety reasons. The implementation of PAT, such as in‐line FTIR (CN_2_ 2091 cm^−1^), is important to monitor that CH_2_N_2_ does not accumulate within the flask.

Our research group, with co‐workers from Patheon, recently described a method for safe handling of CH_2_N_2_ at potentially commercially relevant volumes.^34^ A continuous stirred tank reactor (CSTR) cascade was reported for a modified Arndt–Eistert reaction of *N*‐protected l‐phenylalanine **15** to form diazoketone **16** (Scheme 4 b). A Teflon AF‐2400 membrane was fitted inside each CSTR for the introduction of CH_2_N_2_. After treatment with HCl, α‐chloroketone **17** could be obtained with a throughput of 5.2 mmol h^−1^.

### Trifluoromethyl diazomethane (CF_3_CHN_2_)

**Scheme 5:**
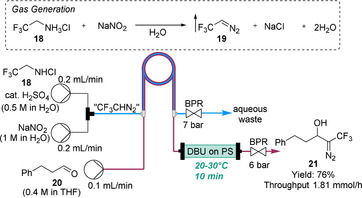
On‐demand generation, separation and reaction of trifluoromethyl diazomethane. DBU=1,8‐diazabicyclo[5.4.0]undec‐7‐ene, PS=polymer support.

Similar to diazomethane, trifluoromethyl diazomethane (CF_3_CHN_2_) (**19**) has both explosive and toxic properties, and is also highly volatile (b.p. 13 °C). It is a highly valuable reagent for the introduction of the trifluoromethyl group. CF_3_CHN_2_ can be prepared from the corresponding amine **18** and aqueous sodium nitrite (NaNO_2_) (Scheme 5).^35^ In the same manner as diazomethane, CF_3_CHN_2_ was observed to pass through the membrane. A yield of CF_3_CHN_2_ with respect to the amine of ≈33 % could be achieved within the tube‐in‐tube reactor, corresponding to 2.5 equiv of the diazo precursor and 5 equiv of NaNO_2_. The continuous generation of CF_3_CHN_2_ was coupled with a cartridge reactor filled with polymer‐supported DBU to perform a base‐catalyzed aldol reaction of aldehyde **20** with CF_3_CHN_2_ to afford compound **21**. The flow protocol was successfully applied to convert a number of aldehydes to their corresponding trifluoromethyl‐functionalized diazo derivatives (nine examples).

### Hydrogen cyanide (HCN)

Hydrogen cyanide (HCN) is important for a number of chemical transformations, including the Strecker reaction for amino acid synthesis, chain elongation of sugars, and hydrocyanation. The main approach used for the preparation of anhydrous HCN is its distillation from aqueous solutions of sodium cyanide (NaCN) or potassium cyanide (KCN) and mineral acid. The use of neat HCN for organic synthesis is limited due to its high toxicity, low boiling point (26 °C) and the possibility of spontaneous exothermic polymerization.

**Scheme 6:**
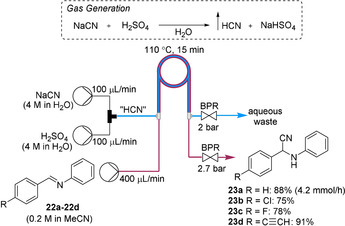
On‐demand HCN generation, separation and reaction for hydrocyanation reactions.

Our group reported the application of a tube‐in‐tube reactor for the in situ generation, separation and reaction of anhydrous HCN.^36^ The system was optimized for the hydrocyanation of diphenylmethaneimine **22 a** (Scheme 6). Aqueous solutions of sodium cyanide (NaCN) and H_2_SO_4_ were pumped to generate HCN. A 2 bar back pressure was applied to prevent out‐gassing of HCN. Full conversion of substrates **22 a**–**22 d** were achieved at 110 °C within 15 min residence time. For slow reactions (reaction times >1 h), a “HCN on tap” configuration was devised whereby the generated and separated anhydrous HCN from the tube‐in‐tube reactor was added in a semi‐batch manner to a round‐bottom flask containing chemistry for the organic transformation. This strategy was important for performing asymmetric reactions, which involve low temperatures (<0 °C), such as an asymmetric Stecker reaction for the preparation of α‐aminonitriles. It also enabled organic transformations involving solids to be conducted. This “gas on tap” strategy could also be applied to the other gases discussed in this Concept. However, flow rates should be carefully controlled to minimize the accumulation of hazardous gas.

### Ammonia (NH_3_)

Ammonia (NH_3_) at high concentrations in both its gas and liquid forms is toxic, corrosive and potentially explosive. In contrast, aqueous NH_3_ is a safe‐to‐handle and relatively inexpensive source of NH_3_. The main limitation of aqueous NH_3_ is that the presence of water which can be detrimental to the performance of many reactions.

**Scheme 7:**
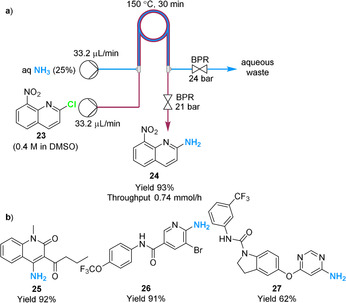
a) Separation of NH_3_ from aqueous solution and ammonolysis of aryl chloride derivatives. b) Complex targets prepared using the protocol from their corresponding aryl chloride.

Zhang, Wu and co‐workers recently reported the use of a tube‐in‐tube reactor for the generation of anhydrous NH_3_ from an aqueous stream of NH_3_ (25 %).^37^ The inner tube contained aqueous NH_3_, whereas the outer tube was the location of the organic reaction. Karl‐Fischer titration analysis determined that the permeation by water was negligible when operating the tube‐in‐tube system at temperatures below 50 °C and shorter residence times. However, it should be noted that at higher operating temperatures the permeation of water was observed. The system was optimized for the nucleophilic substitution on 2‐chloro‐8‐nitroquinoline **23** by NH_3_. An interesting advantage of the protocol is that the system can be operated at 20 bar pressure, which is a higher pressure than accessible by directly using an NH_3_ gas cylinder (typically restricted to approximately 8 bar). The system was successfully applied to the amination of (hetero)aryl fluorides and chlorides to their corresponding primary (hetero)aryl amines (52–97 % yields, 18 examples) over a 10 h production time, including complex intermediates **25**—**27** (Scheme 7).

### Formaldehyde (CH_2_O)

**Scheme 8:**
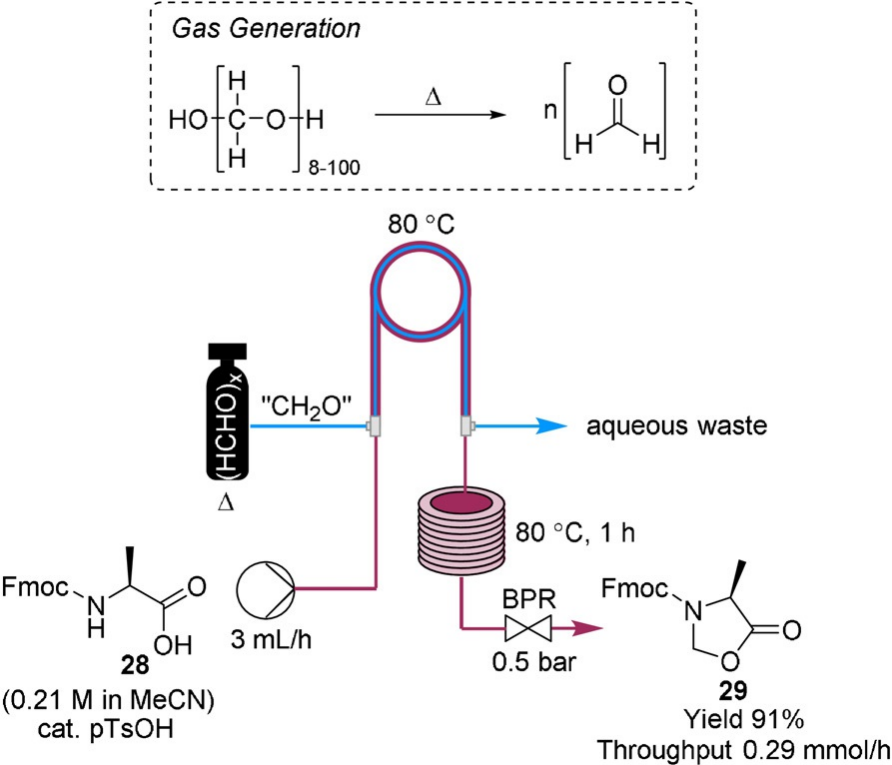
Gaseous formaldehyde is formed in situ from paraformaldehyde and then used for the acid‐catalyzed preparation of oxazolidinone **29** from F‐moc‐l‐alanine **28**. Fmoc=fluorenylmethoxycarbonyl, pTsOH=*para*‐toluenesulfonic acid.

Formaldehyde is used in reactions such as the Cannizzaro reaction, hydroxymethylation and chloromethylation.^38^ It is an adduct form of CO and H_2_ so it can be used as a surrogate to syngas. However, formaldehyde undergoes polymerization below 75 °C, thus its availability in pure form is highly limited. Formaldehyde is predominantly used in its hydrate form as an aqueous solution known as formalin. This solution is corrosive and is not too stable for storage at high or low temperature. Thus, solid paraformaldehyde (HCHO)_*x*_ as the polymer form is more commonly used for its ease to transport, handle, storage and use. An interesting example for in situ generation of formaldehyde was performed by Koch, Kunz and co‐workers.^39^ In this example they heated solid paraformaldehyde (HCHO)_*x*_ within a pressure‐resistant vessel to form gaseous formaldehyde. Paraformaldehyde is depolymerized by heating (80–100 °C). The application of a very low back pressure prevented any out‐gassing. Formaldehyde was used as a reagent in the acid‐catalyzed reaction of Fmoc‐alanine **28** to form oxazolidinone **29**. The tube‐in‐tube reactor was followed by a heater coil to enable the reaction to go to completion to afford compound **29** in 91 % yield within less than 1 h residence time (Scheme 8).

## Challenges and Outlook

The approach for the on‐demand formation of gases from commercially available reagents has been demonstrated for a number of gases. We believe that this Concept can be easily expanded to other gases for organic transformations. For instance, gas–liquid membrane reactors could also be used for the on‐demand generation of anhydrous gases, such as: CO_2_, SO_2_, Cl_2_,^40^ HCl and phosgene (COCl_2_). There is an ever increasing need for the on‐site on‐demand production of chemicals, in particular given problems in securing supply chains for important chemicals and pharmaceuticals.

The aforementioned membrane strategies for on‐demand gas generation are appropriate options for research‐scale experimentation; however, all the approaches currently suffer from limited scalability. All of the protocols discussed are limited by the maximum achievable throughput and by poorer performance at larger scales. Jensen and co‐worker developed a quantitative model to analyze the mass transfer within a tube‐in‐tube reactor.^41^ In this study, they demonstrated that there are many challenges for upscaling the tube‐in‐tube reactor. One possible approach for scale‐up is through a numbering‐up or parallelization strategy. However, in many instances a scale‐up strategy restricted only to numbering‐up is considered inefficient because it requires an accurate fluid distribution which often cannot achieved. The CSTR cascade described for diazomethane is a step toward achieving improved scale‐up, but the demonstrated scale still lacked sufficient throughput to achieve production scale quantities. Thus, an ongoing goal of this research area should be the development of continuous‐flow membrane systems for the on‐demand generation of a gas at any production scale.

The membranes at larger scales become simply too cost prohibitive. Teflon (AF‐2400) has the disadvantage that it is impractical at manufacturing scales due to its high cost ($25 000 kg^−1^).^42^ A relatively inexpensive ($2–10 kg^−1^) fluoropolymer poly(tetrafluoroethylene) (PTFE) membrane has recently been reported,^42^ but its characteristics are not as well understood. Another limitation of Teflon AF‐2400 is that it is very fragile.^43^ In the case of the tube‐in‐tube reactor the fragile inner membrane tubing is protected by the outer tube. In case of fouling then the membrane can be washed by using an appropriate method.^32^ The most common cause of breakage is when a high pressure gradient is applied across the membrane. There is a higher likelihood of failure if the pressure in the outer channel is marginally higher than the inner channel. Thus, careful monitoring of the pressure on both sides of the membrane can extend the membrane lifespan. Furthermore, the use of a 2D flat membrane configuration is reported to extend the membrane lifespan by minimizing failure during operation.^44^ An interesting potentially scalable membrane design was recently reported, whereby a perfluorinated membrane is coated on a hollow fiber made of a thermally and chemically resistant material that provides structural integrity.^45^ The identification of new, low cost membranes, is critical for moving forward. In particular, membrane materials that show excellent chemical compatibility, do not break easily, whilst at the same time allow gas diffusion but are water impermeable. We are currently performing membrane screening experiments within our laboratory to identify potential membrane systems.

Evidently, the handling of hazardous gases needs to be carefully monitored and controlled. The in‐line reaction monitoring of gas generation and reaction should also be implemented more frequently. In terms of process safety, it is important to minimize any accumulation of hazardous gas within the system. The inclusion of process analytical technologies (PAT) will be of increasing importance for the monitoring and control of gas formation and consumption to ensure safety.^46^

## Conclusions

We have outlined an approach for the safe on‐demand generation, separation and reaction of a number of gases from commercially available reagents. Micro‐ and tubular reactors facilitate the generation of small quantities of a gas at any one time. The approach described avoids the main problem associated with in situ gas formation for organic synthesis, whereby the chemistry necessary for the gas release needs to be compatible with the chemistry of the organic transformation. The use of membrane technologies enables the separation of the gas from the reaction conditions and chemicals used for its formation. The organic transformation can then occur within a second chamber. The gas is subsequently consumed rapidly thus preventing the accumulation of a gas. This strategy completely avoids the storage and transportation of hazardous gases. It also drastically improves the safety and circumvents the need for distillation in cases where the gas is generally synthesized in situ, such as diazomethane and HCN. We are convinced that the concept described herein will be embraced by the community thus increasing the use of “difficult‐to‐handle” gases for organic synthesis into the future.

## Conflict of interest

The authors declare no conflict of interest.
